# Assessing the Different Levels of Virtual Reality That Influence Anxiety, Behavior, and Oral Health Status in Preschool Children: Randomized Controlled Clinical Trial

**DOI:** 10.2196/35415

**Published:** 2022-04-18

**Authors:** Naser Asl Aminabadi, Ozra Golsanamlou, Zohreh Halimi, Zahra Jamali

**Affiliations:** 1 Faculty of Dentistry Tabriz University of Medical Sciences Tabriz Iran

**Keywords:** virtual reality, anxiety, behavior, oral health training

## Abstract

**Background:**

Compared with a traditional behavior management strategy and oral health training, virtual reality (VR) integrated with multisensory feedback possesses potential advantages in dentistry.

**Objective:**

This study aimed to assess the impact of different levels of VR on anxiety, behavior, and oral health status.

**Methods:**

This study was carried out in the Department of Pediatric Dentistry at the Tabriz University of Medical Sciences from December 2020 to June 2021. We randomly assigned 60 healthy children aged 4 years to 6 years to 4 groups, each consisting of 15 children. The study consisted of 2 consecutive sessions. During the first visit, the plaque index was calculated, and oral health education was carried out in all groups using Immersive VR (group I), Semi-immersive VR (group II), Nonimmersive VR (group III), and tell-show-do (TSD; group IV). In the second session, an amalgam restoration was performed in all groups. Participants’ anxiety and behavior were recorded using the face version of the Modified Child Dental Anxiety Scale (MCDAS[f]) and Frankl scale. The plaque index was recorded in 2 follow-up sessions.

**Results:**

The greatest prevalence of positive behavior (*P*=.004) and the lowest anxiety (*P*<.001) were recorded in group I, followed by group II, group III, and group IV. The plaque index scores showed a reduced trend between the first session and follow-up sessions (*P*<.001), but the values did not differ significantly between the 4 groups during the 3 sessions (*P*=.28, *P*=.54, *P*=.18).

**Conclusions:**

The most positive behavior was observed in the Immersive VR group, followed by the Semi-immersive VR, Nonimmersive VR, and TSD groups. Moreover, oral health education using VR resources can improve oral health status in children.

**Trial Registration:**

Iranian Registry of Clinical Trials 20210103049926N1; https://www.irct.ir/trial/53475

## Introduction

Virtual reality (VR) is defined as a highly interactive, computer-based multimedia environment in which the user is involved in a computer-generated world [1]. A real or imagined environment can be delivered visually in the 3 dimensions of width, height, and depth, which could additionally provide an interactive experience visually in full real-time motion with sound and possibly with tactile and other forms of feedback [2]. The different types of VR systems that use various technology perform different functions.

Nonimmersive VR systems are the least implemented VR techniques. They involve implementing VR on a desktop computer. Using the desktop system, the virtual environment is observed through a portal by utilizing a standard monitor [3]. A semi-immersive VR system is comprised of a relatively high-performance graphics computing system along with a large screen monitor or multiple television projection systems that increase the depth of immersion [2]. An immersive VR system is the most direct experience of virtual environments in which the user wears a head-mounted display (HMD) to view the virtual environment. An HMD uses small monitors placed in front of each eye that provide stereo, bi-ocular or monocular images [2]. This type of VR system covers the audio and visual perception, cuts out all outside information, and therefore provides a fully immersive visual experience for the observer [4].

From a technological perspective, VR is a set of the following technologies: a helmet, trackers, and a 3D visualizing system. However, from a psychological point of view, VR is simultaneously a simulative technology, a cognitive technology, and an embodied technology. VR is a kind of reality simulation. Specifically, what distinguishes VR from other media is the sense of presence and immersion: the sense of “being there” inside the virtual environment produced by the technology. The simulative power of VR makes it a great tool for experiential learning. On the one hand, VR allows patients to learn through reflection on doing. On the other hand, VR can be described as an advanced imaginative system or an experiential form of imagery that is as effective as reality at inducing emotional responses [5].

A review of the literature revealed evidence of the usefulness of VR technologies for different medical procedures including traumatic injuries, injection or blood sampling, burn care, physiotherapy, and chemotherapy [6-9].

Numerous investigations have extensively addressed the use of immersive VR in dental settings to reduce anxiety and pain during the procedure [10-12]. Use of VR offers a theory-driven approach to educate and train health care providers. The application of a VR technique relies on psychological elements in pain perception. The redirection of attention away from the noxious stimulus, that is distraction, and sensory focusing reduce the severity of the physical injury [13].Moreover, it has been shown that VR engages the patient’s conscious attention and thereby, results in less pain perception [14]. Therefore, redirection of attention modifies internal thoughts by diverting from the real, external environment through immersion in a virtual world by introducing a pleasant experience while engaging higher cognitive and emotional centers of the nervous system.

In addition, current evidence shows that the oral health competency and practice of preschool children were less than adequate [15]. Oral hygiene instructions using educational lectures significantly improve oral health status [16]. However, current evidence suggests that the development of verbal command comprehension skills in preschool children continues for several years, which could explain the difficulty found in the training and practice of oral hygiene techniques using only verbal instructions in this age range [17]. As a result, play-based and audiovisual oral health education has been developed to modify behavioral change and promote tooth brushing skills in children [15].

To the best of our knowledge, there has been no study investigating the impact of different delivery systems of VR on anxiety level, behavior, and oral health education. Therefore, considering the promising profile reported in the literature on the potential impact of VR in children, this study aimed to assess the effect of different levels of VR including nonimmersive VR, semi-immersive VR, and immersive VR in comparison with a conventional behavior management and training strategy on behavior, anxiety, and oral health status of children aged 4 years to 6 years.

## Methods

### Ethical Review

This clinical trial was reported based on the CONSORT (Consolidated Standards of Reporting Trials) statement [18]. Ethical approval for the study was obtained from the Research Ethics Committee of Tabriz University of Medical Sciences (IR.TBZMED.REC.1400.292).

### Recruitment

This study was carried out and funded by the Department of Pediatric Dentistry, Tabriz University of Medical Sciences. The participants consisted of 60 healthy children between 4 years and 6 years of age who attended the Department of Pediatric Dentistry for routine dental treatment from December 2020 to June 2021.

### Sample Size

In the study by Niharika et al [19], which showed a significant decrease in pain perception and state anxiety scores with the use of VR eyeglasses during dental treatment in children aged between 4 years and 8 years, the mean anxiety scores in the intervention and control groups were 14.72 (SD 3.57) and 19.56 (SD 3.74), respectively. Considering an α of .05 and power of 80%, a minimum sample size of 44 was determined. Assuming a dropout rate of about 25%, the minimum calculated sample size was at least 60 patients (15 in each group) to increase the validity of the study.

### Eligibility Criteria

At the first attendance, the Screen for Child Anxiety Related Disorders (SCARED) questionnaire was used to identify patients with anxiety disorders. A total of 60 healthy children aged 4 years to 6 years with nonanxiety disorder was included in the study. Other inclusion criteria were children with no history of invasive medical and dental treatment and the presence of at least one carious mandibular primary molar requiring amalgam restoration.

### Randomization and Blinding

Participants were randomly assigned to 4 groups using the RandList software (DatInf GmbH, Tübingen, Germany). Unique blind codes were used to identify the interventions to blind the outcome assessors and data statisticians.

### Clinical Procedure

Before starting the clinical procedure, written consent was obtained from the parents or legal guardians of the children. All dental procedures in all groups were carried out by a final-year postgraduate pediatric dentistry student. The study consisted of 2 consecutive treatment sessions and 2 follow-up sessions. In the first session, the instruments were introduced to the child using the conventional behavioral control technique (tell-show-do [TSD]) to efficiently establish the child's communication level. The plaque index was calculated using plaque disclosing tablets and recorded as the child’s initial oral health status. Then, oral hygiene instructions were given in all groups. Oral hygiene instructions were provided using an HMD (Immersive VR group; i-glasses 920HR, Ilixco Inc, Menlo Park, CA) in group I, a large television (Semi-immersive VR group; webOS TV, LH590V, LG, Seoul, South Korea) in group II, a tablet (Nonimmersive VR group; Galaxy Tab A7 Lite, Samsung, Seoul, South Korea) in group III, and TSD in group IV. The oral hygiene instructions were provided using VR glasses, on which oral hygiene instructions were demonstrated. The VR used in this study was a passive environment where users were able to visualize in a virtual environment (static not dynamic) with which children could not interact. In the 3 VR groups, the same animation presenting the brushing technique was displayed to the corresponding groups, while moulage scenario training using TSD was used in group IV. A horizontal scrub brushing technique was taught to the children and their parents. Participants were required to brush their teeth twice a day, in the morning and at night before going to bed, for 2 minutes with their parent’s supervision. New sets of toothpaste (Colgate Minions, 0.24% sodium fluoride) and toothbrush (Colgate Kids Toothbrush) were delivered to each parent/child pair. In addition, the parents were instructed to use a “pea-sized” amount of the toothpaste.

In the second session, which took place 1 week after the first session, dental treatment was performed in all 4 groups in addition to the oral hygiene instructions via immersive VR (HMD), semi-immersive VR (large television), nonimmersive VR (tablet), or TSD. In this session, the VR device was introduced to the participants in the VR groups before treatment, and once the VR device was placed on the child’s eyes, the cartoon was started. Then, a topical anesthetic agent was placed on the injection site using a piece of cotton roll, and an inferior alveolar block injection was administered, followed by a class I or II amalgam restoration. Participants in the TSD group received similar procedures without the use of VR distraction.

During the second session, an episode of the Tom and Jerry cartoon was displayed for all 3 VR groups. The participants’ anxiety was measured using the face version of the Modified Child Dental Anxiety Scale (MCDAS[f]), and overall behavior was recorded using the Frankl classification scale. Oral health status was re-examined in 2 follow-up sessions (1 month apart) using the plaque index.

### Instruments

#### SCARED Questionnaire

The parent version of the SCARED questionnaire was designed to assess anxiety symptoms in children under 8 years of age. In this questionnaire, a total score higher than 25 indicated childhood anxiety disorders and therefore were excluded from the present study [20].

#### Face Version of the MCDAS(f) Questionnaire

**Figure 1:**
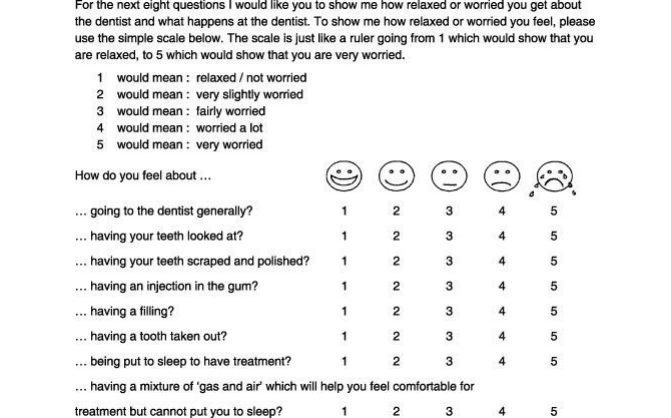
Face version of the Modified Child Dental Anxiety Scale (MCDAS) [22].

This questionnaire is used to evaluate state anxiety in children during conventional dental procedures. This self-report scale consists of 8 questions with 5 pictorial answers for each question. Scores on the MCDAS(f) scale range from 8 to 40, with scores below 19 indicating no state anxiety, scores above 19 indicating state anxiety, and scores above 31 signifying severe phobic disorder (Figure 1) [21].

#### Frankl Behavior Rating Scale

This scale divides observed behavior into the following 4 categories: Rating 1, definitely negative (refusal of treatment, forceful crying, fearfulness, or any other overt evidence of extreme negativism); Rating 2, negative (reluctance to accept treatment, uncooperativeness, some evidence of negative attitude but not pronounced); Rating 3, positive (acceptance of treatment; cautious behavior at times; willingness to comply with the dentist, at times with reservation, but patient follows the dentist’s directions cooperatively); and Rating 4, definitely positive (good rapport with the dentist, interest in the dental procedures, laughter and enjoyment) [23].

### Statistical Analysis

All data were analyzed using SPSS (IBM Corp, Armonk, NY). The results are reported using descriptive statistical analysis (mean [SD] and percentages). Chi-square tests were used to assess gender differences and behaviors between the groups. A one-way analysis of variance (ANOVA) was used to compare the mean plaque index between groups. A Kruskal-Wallis test was used to compare mean MCDAS(f) anxiety scales, mean ages, and mean SCARED scores. A Mann-Whitney *U* test was used to compare mean MCDAS(f) anxiety scales between groups. Repeated measure analysis was performed to compare the mean plaque index in different groups and between different time intervals. The statistical significance was set to .05.

## Results

### Demographic and SCARED Score Results

The final sample consisted of 60 children in 4 groups. There were no statistically significant differences between groups regarding gender (*P*=.86) and age (*P*=.76). The SCARED scores also did not differ significantly between the 4 groups (*P*=.99; Table 1).

**Table 1:** Participants’ gender, age, and Screen for Child Anxiety Related Disorders (SCARED) scores for the 4 groups (total n=60).

Characteristic		TSD^a^ (n=15)	Immersive VR^b^ (n=15)	Semi-immersive VR (n=15)	Nonimmersive VR (n=15)	*P* value
**Gender, n (%)**						
	Female	8 (13)	9 (15)	7 (12)	7 (12)	.87
	Male	7 (12)	6 (10)	8 (13)	8 (13)	
Age (years), mean (SD)		5.25 (0.77)	5.46 (0.63)	5.26 (0.79)	5.21 (0.81)	.76
SCARED score, mean (SD)		12.81 (7.79)	12.66 (7.09)	13.06 (7.88)	12.64 (7.41)	.99

^a^TSD: tell-show-do.

^b^VR: virtual reality.

### Children’s Anxiety Assessment Results

The overall mean MCDAS(f) anxiety score of the participants was 12.68 (SD 4.18). The mean MCDAS(f) anxiety scores of the VR groups and control (TSD) group are shown in Table 2. A statistically significant difference was detected in mean MCDAS(f) scores between the 4 groups (*P*<.001). The lowest MCDAS(f) score was observed in the Immersive VR group, followed by the Semi-immersive VR, Nonimmersive VR, and TSD groups.

Comparison of the mean MCDAS(f) anxiety scores between groups according to the Mann-Whitney *U* test showed statistically significant differences between the 3 VR groups and TSD group. Furthermore, statistically significant differences were detected among the VR groups (Table 3).

**Table 2:** Face version of the Modified Child Dental Anxiety Scale (MCDAS[f]) anxiety scores for the 4 groups.

MCDAS(f)	TSD^a^, mean (SD)	Non-immersive VR^b^, mean (SD)	Semi-immersive VR, mean (SD)	Immersive VR, mean (SD)	*P* value
Anxiety	16.93 (3.61)	14.20 (2.65)	11.33 (2.52)	8.26 (1.57)	<.001

^a^TSD: tell-show-do.

^b^VR: virtual reality.

**Table 3:** Comparison of the face version of the Modified Child Dental Anxiety Scale (MCDAS[f]) anxiety scores between the groups.

Group		Nonimmersive VR^a^	Semi-immersive VR	Immersive VR	TSD^b^
**Nonimmersive VR**					
	*U*	—^c^	47	3.5	59.5
	*P* value	—	.006	<.001	.03
**Semi-immersive VR**					
	*U*	47	—	32.5	24.5
	*P* value	.006	—	<.001	<.001
**Immersive VR**					
	*U*	3.5	32.5	—	5.5
	*P* value	<.001	<.001	—	<.001
**TSD**					
	*U*	59.5	24.5	5.5	—
	*P* value	.03	<.001	<.001	—

^a^VR: virtual reality.

^b^TSD: tell-show-do.

^c^Not applicable.

### Children’s Behavioral Assessment Results

Differences in Frankl scale scores were statistically significant between the 4 groups (*P*=.004; Table 4). The most positive behavior was observed in the Immersive VR group, followed by the Semi-immersive VR, Nonimmersive VR, and TSD groups.

**Table 4:** Comparison of Frankl behavior scale scores between the 4 groups.

Behavior	TSD^a^, mean (SD)	Nonimmersive VR^b^, mean (SD)	Semi-immersive VR, mean (SD)	Immersive VR, mean (SD)	*P* value
Definitely negative	4 (6.7)	1 (1.7)	0	0	.004
Negative	5.8 (8.3)	6 (10)	3 (5)	2 (3.3)	
Positive	6 (10)	8 (13.3)	10 (16.7)	7 (11.7)	
Definitely positive	0	0	2 (3.3)	6 (10)	

^a^TSD: tell-show-do.

^b^VR: virtual reality.

### Oral Health Status Results

The overall mean plaque index scores were 0.71 (SD 0.14), 0.51 (SD 0.15), and 0.49 (0.16) in the first session and first and second follow-up visits, respectively. The mean plaque index scores for all groups are shown in Table 5. There were no statistically significant differences between groups in the first session or first and second follow-up visits (*P*=.28, *P*=.54, and *P*=.18, respectively).

**Figure 2:**
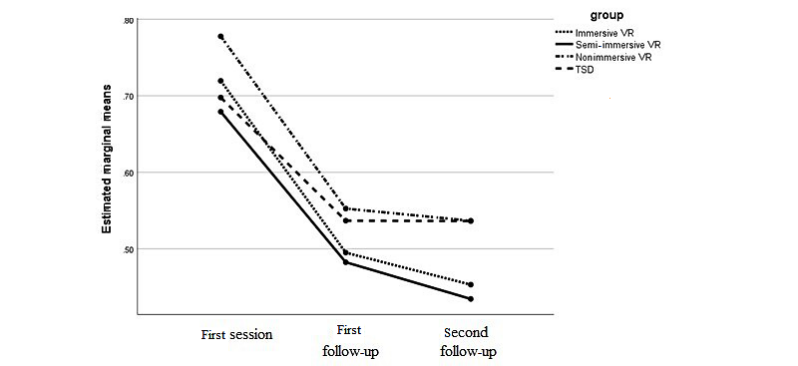
Plaque index scores at each visit for the 4 groups. TSD: tell-show-do; VR: virtual reality.

In addition, repeated measures analysis was performed to compare mean plaque index scores between the different time intervals within each group. Differences in the plaque index scores were statistically significant between the initial session and follow-up sessions in all 4 groups due to the significant sphericity (*P*<.001). However, the interaction between time and group was not significant (*P*=.21; Table 5, Figure 2).

**Table 5:** Plaque index scores at each visit for the 4 groups.

Plaque index	TSD^a,b^, mean (SD)	Nonimmersive VR^b,c^, mean (SD)	Semi-immersive VR^b^, mean (SD)	Immersive VR^b^, mean (SD)	*P* value
Initial session	0.69 (0.19)	0.77 (0.12)	0.67 (0.14)	0.71 (0.09)	.28
First follow-up visit	0.53 (0.17)	0.55 (0.14)	0.48 (0.15)	0.49 (0.12)	.54
Second follow-up visit	0.53 (0.19)	0.53 (0.15)	0.43 (0.16)	0.45 (0.11)	.18

^a^TSD: tell-show-do.

^b^Difference between visits: *P*<.001.

^c^VR: virtual reality.

## Discussion

### Principal Findings

This study showed that the lowest anxiety score and the most positive behavior were observed in the Immersive VR group, followed by the Semi-immersive VR, Nonimmersive VR, and TSD groups. The results revealed that there were no statistically significant differences in mean plaque index scores between the 4 groups.

In this randomized clinical trial, we evaluated the impact of different levels (delivery systems) of VR on anxiety and behavior in children during the dental procedures. In addition, this study aimed to evaluate the impact of oral health education using different levels of VR on children’s oral hygiene status.

All participants underwent a rigorous screening process using the parent version of the SCARED questionnaire to exclude the children with trait anxiety disorders, which is defined as a general predilection to respond with anxiety in threatening situations including dental procedures. Moreover, since a history of painful medical or dental experiences has been identified as an important determinant of anxiety and pain perception, in this study, participants with a history of any dental and surgical procedures were excluded [24]. Meanwhile, it has been suggested that using favorite, familiar, fantasy children’s characters could prevent them from focusing on the anxiety-inducing appearance of dental equipment; therefore, a similar episode of the Tom and Jerry cartoon series was played for all patients [25].

### Effects of VR on Children’s Anxiety and Behavior

The results of this study revealed that different VR delivery systems were effective in decreasing anxiety during the dental procedure. The lowest prevalence of anxiety was experienced by the children in the Immersive VR group, followed by the Semi-immersive VR, Nonimmersive VR, and TSD groups. Furthermore, the VR application induced more positive behavior in children during routine dental procedures. These results represent significant practical improvements in behavioral outcomes in the Immersive VR group, followed by the Semi-immersive VR, Nonimmersive VR, and TSD groups. These results suggest that, with increasing immersion depth, children’s attention will be pulled more from the real world, and thereby, children experience lower levels of dental anxiety and more positive behaviors.

The application of VR is based on the psychological theories of pain perception in which anxiety and pain are the expressions of sensory inputs; therefore, the cognitive appraisal of emotions is important in the process and degree of stress experienced by the patients [26]. The hypothesis that distraction reduces distress is clearly relying on cognitive models, and it is assumed that the experience of distress depends on information processing. Anxiety is caused by paying attention to sensory inputs and processing them emotionally, thus distraction could interrupt this process and reduce pain perception [26].

Furthermore, as demonstrated by Kahneman’s capacity model, individuals have a limited pool of information-processing resources, and using their capacity for one specific activity limits their availability for other activities [27]. Thus, engaging in an attention-grabbing activity confuses available attention and prevents the processing and accessing of other information. It seems that VR robs precious cognitive resources from other information-processing activities such as dental procedures. In a similar context, increasing the level of immersion using multisensory VR (visual, auditory, and sometimes tactile elements) creates significant cognitive demand on patients and therefore steals cognitive resources from other events. Thus, more immersive VR could exert a more distractive effect by diverting conscious attention away from painful and anxious stimulation, leading to reduced subjective pain and anxiety levels [13,14]. It is not surprising that complete blockage of the child’s visual fields and providing audiovisual inputs via the VR eyeglasses in the Immersive VR group resulted in the lowest level of anxiety and the most positive behavior.

On the other hand, new research attempts to monitor brain changes using functional magnetic resonance imaging (fMRI) during VR device use [28]. This finding shows a strong relationship between the neurological and psychological components of pain; when a person pays less attention to pain, pain severity in the brain will decrease. With the use of VR devices, not only did the participants report reduced pain but their fMRI scans also showed a reduction in pain-related brain areas including the primary and secondary somatosensory cortex, insula, thalamus, and anterior cingulate cortex. Therefore, the users of VR devices not only experience audiovisual distraction but also exhibit decreased neural activity in pain-related brain regions [28].

In accordance with results of this study, a review of the literature revealed a reduction in pain and anxiety levels in the majority of studies using VR devices during dental procedures [10,11,28]. Moreover, findings of this study are also consistent with the evidence of prior studies on the relationship between VR and different medical procedures including intravenous (IV) placement, chemotherapy, and physiotherapy [29-31].

However, the results of this study differ from the study conducted by Alhalabi et al [10], in which using VR eyeglasses had no significant effect on anxiety and pain perception during inferior alveolar nerve block injection in 6‒10-year-old children. Part of this inconsistency could be attributed to the different populations and parameters collected and analyzed. In the study by Alhalabi et al [10], discomfort was evaluated just after administering the inferior alveolar nerve block, while in other studies, anxiety and pain were recorded during the entire treatment procedure. Further, differences in the age ranges of the children could explain the underlying contradictory results. The findings of the study by Das et al [32] provide supportive evidence for our line of reasoning that older children suppose VR technology to be a simple game, while younger consumers are significantly captivated by VR's immersive power in engaging children and harnessing their emotions [32]. Since coping skills and cognitive ability are underdeveloped in preschool children, distraction techniques including VR is a crucial part of a behavior management strategy. Therefore, it is not surprising that a VR technique was more effective in preschool children in comparison with older children [32].

In addition, in the study by Alhalabi et al [10], a very large VR device was used. Therefore, the practitioner’s vision was considerably blocked during the dental procedures and local anesthesia administration, which might explain the differences in the practitioner’s understanding of the clinical situation. In this study, appropriately sized VR eyeglasses were used to accommodate the size of children while completely blocking the participant’s vision.

In accordance with the results of this study, the findings of studies using a crossover design to test VR efficacy have confirmed reduced pain perception and anxiety levels in healthy children during 2 consecutive dental sessions [12,33]. The advantage of a crossover study is that each participant would be compared to themselves in both experimental and control situations.

It is worth noting that, although positive behaviors and reduced anxiety levels were observed in the Nonimmersive and Semi-immersive groups, the lowest anxiety level and the most positive behavior were seen in the Immersive VR group. These advantages are attributed to the complete blockage of the child’s visual field and greater immersion of the child by application of immersive VR devices.

### Effects of VR on Children’s Oral Hygiene Status

The second aim of this study was to examine the effect of oral health education using different VR delivery systems on oral health promotion in 4–6-year-old children.

Although the result of this study revealed a reduced plaque index in all studied groups, the difference did not reach a statistically significant level. In contrast with the results of this study, Chang et al [15] suggested a digital design for tooth brushing with UbiComp technology to promote the brushing skills of kindergarten children. They reported that this technology promotes tooth brushing skills of children in a short training time and can significantly improve children’s oral health status.

Nonsignificant differences between plaque indexes in the VR and control groups can be attributed to the fact that a mouth moulage was used to demonstrate tooth brushing to the participants in the control group, which was more effective than verbal commands and tangible for the child. However, using VR to educate about oral hygiene practices and tooth brushing in VR groups was also interesting for the children and their parents. It should be noted that virtual realism may be related with static or dynamic objects [34]. In this study, users were able to visualize in a virtual environment (static), but in the studies focused on digital methods, users were able to visualize and interact in a virtual environment (dynamic) [15]. Furthermore, virtual environments employ augmented reality (AR) as a learning tool and provide a more realistic experience for the participants. AR is a technology that superimposes a computer-generated virtual scenario atop an existing reality in order to create a sensory perception through the ability to interact with it; therefore, AR seems to be more effective in real operations than VR [35]. In this study, due to the application of audiovisual systems of VR without a haptic tracker or AR technique for oral health education, no difference was observed between the different VR delivery systems as well as between VR and the control group.

Despite reports of simulation sickness, nausea, and eye strain in young children using the VR technique, the participants in our study did not have any adverse effects nor discomfort using the VR [7].

Although this study offers a clear picture of the influence of different VR delivery systems on anxiety, behavior, and oral health education in preschool children, the findings should be considered in the context of some limitations. One of the limitations in this study is the age of the participants, which makes it difficult to generalize the findings to other age groups. Since different age groups exhibit different cognitive characteristics and behavioral patterns toward the VR technique, conducting prospective research studies utilizing different age groups is highly recommended for future studies. In addition, we suggest considering mediating factors influencing VR, including the different types of software and hardware of VR devices. Furthermore, our findings endorse the necessity of conducting studies using various medical and dental procedures and environments. Moreover, further studies addressing other preventive measures of oral hygiene practice are suggested.

### Conclusion

Based on the obtained results, the lowest anxiety score and the most positive behavior were observed in the Immersive VR group, followed by the Semi-immersive VR, Nonimmersive VR, and TSD groups. However, the results did not show statistically significant differences in mean plaque index scores between the 4 groups at the first and second follow-up visits. Therefore, it can be concluded that different levels of VR can be effectively used to reduce anxiety and promote positive behavior during routine dental procedures. Moreover, oral health education using VR resources can improve oral health status in children; however, using traditional methods of education would result in a comparable rate of improvement in oral health condition.

## Appendix

Multimedia Appendix 1 CONSORT-eHEALTH checklist (V 1.6.1).

## Abbreviations

ANOVA: analysis of variance
AR: augmented reality
CONSORT: Consolidated Standards of Reporting Trials
fMRI: functional magnetic resonance imaging
HMD: head-mounted display
IV: intravenous
MCDAS(f): face version of the Modified Child Dental Anxiety Scale
SCARED: Screen for Child Anxiety Related Disorders
TSD: tell-show-do
VR: virtual reality
